# Surgical aortic valve replacement in patients older than 75 years: is there really a quality of life benefit?

**DOI:** 10.1007/s12471-015-0660-2

**Published:** 2015-02-21

**Authors:** Charlotte van Laar, Peter C. Kievit, Luc Noyez

**Affiliations:** 1 Department of Cardio-Thoracic Surgery-615, Heart Center, Radboud University Nijmegen Medical Center, PO Box 9101, 6500 HB Nijmegen, The Netherlands; 2 Department of Cardiology-611, Radboud University Nijmegen Medical Center, PO Box 9101, 6500 HB Nijmegen, The Netherlands

**Keywords:** Aortic valve replacement, Elderly, Quality of life, Survival

## Abstract

**Background:**

To evaluate the results of elective isolated surgical aortic valve replacement (SAVR) on quality of life (QoL) in patients > 75 years.

**Methods:**

138 patients operated between January 2008 and December 2011 were included. The EuroQOL questionnaire (EQ-5D, EQ-VAS) was completed preoperatively, and 1- and 2-years postoperatively. The logistic EuroSCORE was used for risk stratification, the Corpus Christi Heart project criteria to assess physical activity.

**Results:**

Mean age was 79.5 ± 2.8 years, mean risk 9.7 ± 5.4, hospital mortality 2.8 %. For 115 patients (83.3 %) the preoperative QoL information was complete. Fifty patients were classified as sedentary. In the first postoperative year 13 patients died, mostly sedentary patients (*p* = 0.046) with a low EQ-5D (*p* = 0.017). There was no QoL information on 32 survivors, mostly sedentary patients (*p* = 0.001). The 70 patients with QoL information showed an increased QoL (NS). Two years postoperatively, 16 patients died, significantly more sedentary patients (*p* = 0.015) with a low EQ-5D (*p* = 0.006). For 42 survivors, there was no QoL information; these were mostly sedentary patients (*p* = 0.021). The 57 patients with 2-year QoL information had an increased EQ-5D (NS) and EQ-VAS (*p* = 0.024).

**Conclusions:**

QoL increases after SAVR. However, the patients lost to follow-up were mostly sedentary or had a low preoperative QoL, which can lead to biased results.

## Introduction

Aortic valve stenosis is the most common acquired native valve disease in Western countries and its prevalence increases with age [1, 2]. Consequently, surgical aortic valve replacement (SAVR) is increasingly performed in elderly patients [1, 2]. In these elderly patients not only the survival and operation-related mortality and morbidity are important, but also the quality of life (QoL) [3, 4]. Essential in the evaluation of QoL post-cardiac surgery is the comparison of preoperative and postoperative QoL. In a previous paper we pointed out the lack of preoperative QoL information in several QoL studies [5]. A recent review concerning QoL benefits after aortic valve surgery in the elderly confirmed that most studies are retrospective and do not compare baseline QoL with postoperative QoL [6]. Generally, QoL at a certain moment in the follow-up is compared with QoL of an aged-matched general population [5, 6]. In this way QoL post-intervention is compared with QoL of a normal population, and not the impact of the intervention on QoL. Moreover, these studies are in a matter of speaking misleading, because they only take into account QoL of patients who survived the follow-up period.

The intention of this study is to evaluate the hospital mortality, major morbidity, survival and QoL of patients older than 75 years undergoing elective isolated SAVR by comparing preoperative baseline QoL with QoL scores at 1 and 2 years after surgery.

## Patients and methods

### Patients

From our Cardiac Surgery Database Radboud Hospital (CORRAD)- a database that stores pre-, per-, and post-operative data plus follow-up data from all adult patients undergoing cardiac surgery at the Radboud University Medical Centre Nijmegen (RadboudUMC) – we identified 427 consecutive patients who underwent isolated SAVR between 1 January 2008 and 31 December 2011. Of these, 142 patients were older than 75 years and 138 of them underwent elective surgery. These 138 patients are our study population.

### Risk stratification and hospital mortality and morbidity

The initial logistic EuroSCORE was used for risk stratification [7]. Hospital mortality was defined as death after cardiac surgery occurring at any time during initial hospital admission in the cardiac surgery centre. Hospital major morbidity was defined as prolonged ventilation, deep sternal infection, permanent stroke, renal failure and reoperation and the composite score- any major morbidity- as defined in the report of the Society of Thoracic Surgeons (STS) Quality Measurement Task Force [8].

### Quality of life registration

To assess QoL both components of the EuroQOL instrument (EQ-5D and EQ-VAS) were used [9]. This is a validated standardised generic instrument to measure QoL. The EQ-5D consists of five domains of health (Mobility, Self-Care, Usual Activities, Pain/Discomfort, and Anxiety/Depression), each of which is divided into three levels: no problems (i), some or moderate problems (ii) and extreme problems (iii). Based on the response to this classification, a single index value is estimated using a general population-based algorithm [10]. The EQ-5D score spans a scale from − 0.54 to 1.0, with greater scores indicating better quality of life. For the EQ-VAS, patients estimate their own health on a visual analogue scale ranging from 0 to 100, with 0 being the worst possible health state and 100 being the best. The EQ-5D index can be regarded as a societal-based composite global QoL measure, whereas the EQ-VAS is a direct global QoL assessment from the patient’s perspective. Patients were asked to individually complete the preoperative QoL questionnaire on the day before surgery, therefore only patients undergoing elective surgery were included in our QoL studies. Important is that only patients with complete preoperative QoL information (both EQ-5D and EQ-VAS) were retained in this part of the study.

Since a majority of patients considered increased mobility post-cardiac surgery to be a very important goal in itself, as well as an aspect of their QoL, several years ago the activity levels of the Corpus Christi Heart Project (CCHP), [11] especially for assessing physical activity, were added to our QoL questionnaire [12]. It must be clear that the intention of this paper is not to study physical activity post-SAVR. However, the fact that we have this preoperative information permits us to classify our patients as sedentary, no physical activity above minimum demands of daily living or light or fairly light activity during normal daily routine, or active patients [11].

### Follow-up

Follow-up is a fixed part of our CORRAD registration and is performed for all patients discharged from the RadboudUMC, in the first, second and fifth postoperative year. A written survey is sent directly to the patients [13, 14]. This survey includes, beside questions concerning survival and events, the same QoL questionnaire as given preoperatively. Participation in the survey is voluntary, no patients were contacted by phone or received a second mailing in this QoL study. However, when recent survival follow-up information (≤ 2 years) was missing, we contacted the patient or general practitioner by phone, leading to a 100 % complete 2-year survival follow-up.

The registration of data in the CORRAD database and the use of this information for research were approved by the local ethics and research council of the Radboud University Nijmegen [13].

### Statistical analysis

Statistical analyses were performed using IBM SPSS Statistics 20, Chicago, IL, USA. Data are presented as percentage for dichotomous variables, and as mean ± standard deviation (SD) and range for numerical variables. In the tables median and interquartile range (IQR) is added for the numerical variables. Differences in characteristics were assessed using the Chi-square test or the Fisher’s exact test for discrete variables and the two-sample Student’s t-test for continuous variables. Statistical significance is assumed at *p* ≤ 0.05.

## Results

The 138 patients in our study had a mean age of 79.5 ± 2.8, (75.1–87.5) years, and 56 (41 %) were older than 80 years. Eighty-four patients were female (61 %).The mean logistic EuroSCORE was 9.7 ± 5.4, (4.4–38.9).

### Morbidity and survival

Major morbidity concerns 11 resternotomies, 3 patients with renal failure and 5 patients with prolonged ventilation. The composite morbidity score was 11.5 % (16/138). Hospital mortality was 2.8 % (*n* = 4). Our 1-year mortality was 9.3 %, as 9 patients died during the first year after hospital stay. In the second postoperative year another 3 patients died. The 1- and 2-year overall survival rates were 90 and 88 %, respectively.

### Quality of life

We received complete preoperative QoL and physical activity information for 115/138 patients (83.3 %). The mean EQ-5D was 0.69 ± 0.27 (− 0.11–1.0) and the mean VAS 58.9 ± 20.6 (0–100). Based on the CCHP criteria, 50 patients (43.8 %) could be classified as sedentary and 65 patients (56.2 %) as active.

No statistically significant differences were detected between the 115 patients with preoperative QoL information and the 23 patients without preoperative QoL information with respect to age, percentage of female patients, logistic EuroSCORE risk, the percentage of morbidity score and hospital mortality (Table 1).

**Table 1:** Baseline characteristics of the population with preoperative QoL information (QoL group) and the population without preoperative QoL information (excluded)

Variable	QoL group *N* = 115	Excluded *N* = 23	*p*-value
Age (years)			0.530
Mean ± SD (range)	79.3 ± 2.9 (75.1–87.5)	79.7 ± 3.4 (75.1–87.6)	
Median [IQR]	79.0 [76.9–81.2]	79.2 [77.7–81.5]	
Female gender	70 (61 %)	14 (57 %)	0.697
Logistic EuroSCORE			0.677
Mean ± SD (range)	9.7 ± 5.0 (4.3–31.6)	10.2 ± 7.1 (6.3–18.9)	
Median [IQR]	7.9 [6.3–11.2]	10.0 [6.4–9.4]	
Morbidity score	14 (12.7 %)	2 (8.6 %)	0.402
Hospital mortality	4 (4.3 %)	0	0.308

*IQR* interquartile range, *QoL* quality of life, *SD* standard deviation

Thirteen of the 115 patients with preoperative QoL information died during the first postoperative year. Table 2 compares age, percentage of female patients, preoperative logistic EuroSCORE risk, preoperative physical activity level, preoperative EQ-5D and EQ-VAS between patients who died and patients who survived the first postoperative year. Patients who died in the first year were more often sedentary (*p* = 0.046) and had a lower preoperative EQ-5D (*p* = 0.017).

**Table 2:** Characteristics of the population with preoperative QoL information (QoL group) and proven 1-year survival versus the QoL group who died within the first postoperative year

Variable	QoL group 1-year survival *N* = 102	QoL group 1-year mortality *N* = 13	*p*-value
Age (years)			0.639
Mean ± SD (range)	79.3 ± 2.9,(75.1–87.5)	79.7 ± 3.4 (75.1–87.4)	
Median [IQR]	78.8 [76.9–81.2]	79.2 [77.7–80.6]	
Female gender	61 (59 %)	9 (69 %)	0.512
PA level: sedentary	41 (40 %)	9 (69 %)	0.046
Logistic EuroSCORE			0.249
Mean ± SD (range)	9.5 ± 5.0 (4.3–31.6)	11.2 ± 4.1 (6.3–18.9)	
Median [IQR]	7.3 [6.2–10.7]	11.0 [7.3–14.1]	
EQ-5D—preoperative			0.017
Mean ± SD (range)	0.71 ± 0.27 (− 0.11–1.0)	0.52 ± 0.29 (0.7–1.0)	
Median [IQR]	0.80 [0.65–0.87]	0.41 [0.29–0.82]	
EQ-VAS—preoperative			0.128
Mean ± SD (range)	59.8 ± 20.3 (0–100)	51.5 ± 17.2 (20–75)	
Median [IQR]	60 [50–75]	50 [40–70]	

*IQR* interquartile range, *PA* physical activity, *QoL* quality of life, *SD* standard deviation

One-year postoperative QoL information was delivered by 102 patients (89 %); however, postoperative QoL information was complete in only of 70 of these patients (69 %). Table 3 presents the group of 70 patients with 1-year QoL information versus the group of 32 patients without QoL information. The proportion of sedentary patients was significantly higher in the group of patients without 1-year QoL information: 66 versus 29 % (*p* = 0.001). The logistic EuroSCORE tended to a higher risk for the group without QoL information 10.8 ± 6.6 versus 8.9 ± 4.1 (*p* = 0.071). The baseline EQ-5D and EQ-VAS values were lower for the patients without 1-year QoL information; however, these differences were not statistically significant.

**Table 3:** Characteristics of the population with preoperative QoL information (QoL group) and proven 1-year survival with QoL information versus the group without QoL information 1-year postoperatively

Variable	QoL group with 1-year survival + QoL information *N* = 70	QoL group with 1-year survival-QoL information *N* = 32	*p*-value
Age (years)			0.287
Mean ± SD (range)	79.3 ± 2.8 (75.1–87.5)	78.8 ± 2.9 (75.1–85.3)	
Median [IQR]	79.9 [77.1–81.4]	78.3 [76.6–80.3]	
Female gender	39 (55 %)	22 (69 %)	0.213
PA level: sedentary	20 (29 %)	21 (66 %)	0.001
Logistic EuroSCORE			0.071
Mean ± SD (range)	8.9 ± 4.1 (4.71–21.53)	10.8 ± 6.6 (4.4–31.6)	
Median [IQR]	7.1 [6.2–10.1]	8.5 [6.5–11.9]	
EQ-5D–preoperative			0.460
Mean ± SD (range)	0.73 ± 0.24 (0.6–1.0)	0.68 ± 0.32 (− 0.11–1.0)	
Median [IQR]	0.79 [0.65–0.84]	0.80 [0.58–0.89]	
EQ_VAS preoperative			0.156
Mean ± SD (range)	61.8 ± 18.8 (0–100)	55.6 ± 22.9 (0–95)	
Median [IQR]	60 [50–76]	60 [40–70]	

*IQR* interquartile range, *PA* physical activity, *QoL* quality of life, *SD* standard deviation

The 70 patients with complete preoperative and 1-year postoperative QoL information showed an increase for the EQ-5D from 0.73 ± 0.24, (0.07–1.00) preoperatively to 0.77 ± 0.23, (0.06–1.00) postoperatively (*p* = 0.274). The EQ-VAS increased from 61.8 ± 18.9, (0–100) preoperatively to 67.4 ± 21.5, (0–100) postoperatively, which tended to significance (*p* = 0.058).

Two years after surgery, 16 patients had died, so only 99 patients were able to deliver information about 2-year postoperative QoL. The 16 patients who died tended to be older (*p* = 0.087), had a higher percentage of sedentary patients (69 versus 39 %, *p* = 0.015), a trend to a lower preoperative EQ-VAS (*p* = 0.087) and a statistically significantly lower EQ-5D (0.51 ± 0.27 versus 0.72 ± 0.27, *p* = 0.006) than the patients who survived the first 2 postoperative years (Table 4).

**Table 4:** Characteristics of the population with preoperative QoL information (QoL group) and proven 2-year survival versus the QoL group who died within the first two postoperative years

Variable	QoL group 2-year survival *N*=99	QoL group 2-year mortality *N* = 16	*p*-value
Age (years)			0.087
Mean ± SD (range)	79.2 ± 2.8 (75.1–86.6)	80.1 ± 3.2 (75.1–87.1)	
Median [IQR]	78.8 [76.9–81.1]	79.1 [78.1–81.9]	
Female gender	59 (60 %)	11 (69 %)	0.486
PA level: sedentary	39 (39 %)	11 (69 %)	0.015
Logistic EuroSCORE			0.176
Mean ± SD (range)	9.4 ± 5.1 (4.3–31.6)	11.3 ± 3.9 (6.3–18.9)	
Median [IQR]	7.2 [6.2–10.7]	11 [7.6–14.5]	
EQ-5D-preoperative			0.006
Mean ± SD (range)	0.72 ± 0.27 (− 0.11–1.0)	0.51 ± 0.27 (0.07–1.0)	
Median [IQR]	0.80 [0.00–0.89]	0.49 [0.25–0.77]	
EQ-VAS–preoperative			0.087
Mean ± SD (range)	60.1 ± 20.1 (0–100)	50.9 ± 18.6 (20–80)	
Median [IQR]	60 [62–80]	50 [40–70]	

*IQR* interquartile range, *PA* physical activity, *QoL* quality of life, *SD* standard deviation

The 2-year postoperative QoL information was complete in only 57 of the 99 patients (58 %). Table 5 shows that there is no statistically significant difference for age, gender ratio, logistic EuroSCORE risk, EQ-5D and EQ-VAS between the patients with or without 2-year QoL information, with the exception of a statistically significantly higher proportion of sedentary patients without 2-year QoL information (52 versus 30 %, *p* = 0.021).

**Table 5:** Characteristics of the population with preoperative Qol information (QoL group) and proven 2-year survival with QoL information versus the group without QoL information 2 years postoperatively

Variable	QoL group with 2-year survival + QoL *N* = 57	Qol group with 2-year survival-QoL *N* = 42	*p*-value
Age (years)			0.543
Mean ± SD (range)	79.4 ± 2.8 (75.1–86.3)	79.0 ± 2.8 (75.1–86.6)	
Median [IQR]	78.7 [77.1–81.3]	78.7 [76.6–80.3]	
Female gender	32 (56 %)	27 (64 %)	0.389
PA level: sedentary	17 (30 %)	22 (52 %)	0.021
Log. EuroSCORE			0.640
Mean ± SD (range)	9.2 ± 5.1 (4.8–30.6)	9.7 ± 5.0 (4,3–31,6)	
Median [IQR]	7.1 [6.1–10.6]	8.2 [6.5–11.8]	
EQ-5D-preoperative			0.879
Mean ± SD (range)	0.72 ± 0.245 (0.0–1.0)	0.72 ± 0.28 (− 0.11–1.0)	
Median [IQR]	0.80 [0.65–0.85]	0.80 [0.65–0.89]	
EQ_VAS-preoperative			0.533
Mean ± SD (range)	59.1 ± 21.5 (0–100)	61.6 ± 18.1 (25–95)	
Median [IQR]	60 [50–75]	60 [40–70]	

*IQR* interquartile range, *PA* physical activity, *QoL* quality of life, *SD* standard deviation

The 57 patients with complete preoperative and 2-year postoperative QoL information showed a trend to an increase for the EQ-5D from 0.72 ± 0.25 (0.80), 0.07–1.00 preoperative to 0.77 ± 0.24 (0.02–1.00) postoperative (*p* = 0.117). The EQ-VAS significantly increased from 59.1 ± 21.5 (0–100) preoperatively to 67.4 ± 21.5 (0–100) postoperatively (*p* = 0.024).

## Discussion

Our study population has a mean age of 79 years and a calculated mean operative risk of 9.7. This risk is comparable with the calculated mean logistic EuroSCORE risk of 8.2 for patients undergoing isolated SAVR in the Netherlands, as described by Siregar et al. [15]. Notably, the study by Siregar et al. concerns patients of all ages and both elective and non-elective operations, whereas our study includes only elective surgery and patients older than 75 years. The same remark concerns our evaluation of the hospital mortality of 2.8 % and a morbidity, composite score of 11.4 versus the 3.1 % (IQR 2.4–4.0 %) and 17.8 % (14.5–21.6 %) as presented by the STS as composite measure for isolated SAVR [8]. These data confirm, as in several other studies, that SAVR can be performed in elderly patients with acceptable hospital mortality and morbidity [6].

The preoperative QoL questionnaire was completed by 83.3 % of the patients. It is already exceptional that we have this preoperative QoL information whereas most studies only have postoperative QoL information [5, 6]. Furthermore, the percentage of 83.3 % is high, even higher than in our overall patient population (71.4 %) [16]. In view of the ongoing (preoperative) discussion between cardiologists, cardiac surgeons and patients about the benefits of the surgery in terms of the postoperative survival and QoL, these patients may be more willing to complete the questionnaire.

We observed no difference for the studied variables between patients who completed the QoL questionnaire and those who did not. Thus, we did not exclude the most fragile patients in the specific QoL evaluation. The preoperative values of EQ-VAS and EQ-5D were comparable with our previous study concerning patients aged 70 years and older [17]. The available information on preoperative physically active levels in our study provides additional insights.

For the evaluation of the QoL 1-year postoperatively we first evaluated the characteristics of the 13 patients who did not survive the first postoperative year. In comparison with the patients who did survive the first year, these patients were at higher risk, although this was not statistically significant. Moreover, the proportion of sedentary patients was significantly higher in the group of patients who did not survive in this group. The EQ-5D and EQ-VAS were significantly and non-significantly lower, respectively. Secondly we evaluated the 32 patients who survived the first postoperative year, but who did not complete their 1-year postoperative QoL questionnaire, the so-called dropouts [5, 16]. In comparison with the patients with complete 1-year QOL information, we see the same trend as in the group of patients who died: higher risk, a significantly higher percentage of sedentary patients and lower preoperative EQ-5D and EQ-VAS. The patients with preoperative and postoperative QoL information show a non-statistically significant increase of the EQ-5D and an increase tending towards significance of the EQ-VAS.

After the second follow-up year we notice the same trends as during the first postoperative year. The patients who died were older and at higher risk, were significantly more often sedentary and had a significantly lower preoperative EQ-5D. Also the percentage of sedentary patients among the dropouts was significantly higher. When comparing preoperative QoL with 2-year postoperative QoL, we found a statistically significant increase in the EQ-VAS, however not in the EQ-5D.

That the increase in QoL is mainly due to the more subjective patient perspective (EQ-VAS) was also observed in our previous study [17]. Other reports confirm good QoL post SAVR in the elderly; however, most of these studies were based on comparison of a patient population that survived SAVR with an age- and sex-matched general population [6]. Our study confirms also and again, that missing data in QoL studies are not at random [18]. Also in patients undergoing transcatheter aortic valve replacement (TAVI), where QoL is an important goal, [19] we have the same problem. In the systematic review published by Shan et al., only one report concerning TAVI patients compares really preoperative QoL versus postoperative QoL [20].

Our study has some limitations. First, our study is a single-centre experience and concerns a limited patient population. Second, the EuroQOL is not frequently used in cardiac surgery research; however, it is a validated and internationally accepted scoring system for registration of quality of life.

Based on our results, we conclude that SAVR can be performed in patients older than 75 years with an acceptable hospital mortality, morbidity and short-term survival. QoL increases after SAVR, patients feel better and subjectively experience a better quality of life. However, over time several patients are lost for QoL evaluation, and the results in these patients may be of particular interest.
